# Mutations Targeted by Nous-209 Immunotherapy Occur Early in Lynch Syndrome Carriers’ Precancer Lesions with Microsatellite Instability

**DOI:** 10.1158/1940-6207.CAPR-25-0388

**Published:** 2026-05-08

**Authors:** Elisa Micarelli, Lorenzo De Marco, Paola Spaggiari, Anna Morena D’Alise, Arianna Dal Buono, Maddalena Menini, Valentina Giatti, Alessandro D’Aprano, Elisa Scarselli, Cesare Hassan, Alessandro Repici

**Affiliations:** 1 Nouscom SRL, Rome, Italy.; 2 Department of Pathology, IRCCS Humanitas Research Hospital, Rozzano, Milan, Italy.; 3 Department of Gastroenterology, IRCCS Humanitas Research Hospital, Rozzano, Milan, Italy.; 4 Department of Biomedical Sciences, Humanitas University, Pieve Emanuele, Milan, Italy.

## Abstract

This study provides a molecular characterization of precancerous colorectal lesions in Lynch syndrome (LS) carriers to assess the preventive potential of Nous-209 immunotherapy against colorectal cancer development. A total of 50 adenomas and 12 advanced adenomas (AA) were collected from 26 LS carriers with pathogenic variants in either *MLH1* or *MSH2*. Molecular analyses included assessment of mismatch repair (MMR) status, microsatellite instability (MSI), and detection of mutations targeted by Nous-209. We found that 83% of AAs and 58% of adenomas were MMR-deficient (dMMR). Notably, although all dMMR AA were MSI-high (MSI-H), only 66% of dMMR adenomas showed MSI-H. The presence of Nous-209 mutations correlated strongly with MSI status, with mutation counts ranging from 15 to 57 in dMMR/MSI-H lesions. dMMR adenomas classified as MSI-low carried a limited number of mutations (6–19), whereas microsatellite-stable lesions harbored very few (0–2) Nous-209 mutations, regardless of MMR proficiency. These findings confirm the molecular heterogeneity of precancerous lesions and support the potential of Nous-209 immunotherapy to prevent MSI colorectal cancer in LS by targeting the adenoma–carcinoma sequence at the time of MSI acquisition.

**Prevention Relevance:**

Our study shows that MSI and neoantigen accumulation emerge during the evolution of precancerous lesions in LS. These findings support the clinical evaluation of Nous-209, a shared neoantigen vaccine, as an immunoprevention strategy for MSI-driven colorectal carcinogenesis, with important implications for cancer prevention research.

## Introduction

Lynch syndrome (LS) arise from heterozygous germline pathogenic variants in one of the DNA mismatch repair (MMR) genes—*MLH1*, *MSH2*, *MSH6*, or *PMS2*—or from *EPCAM* deletions leading to *MSH2* silencing (1). LS affects up to 1 in 279 individuals and predisposes carriers to cancers with microsatellite instability (MSI; refs. 2, 3), most notably colorectal and endometrial cancers, though increased risk extends to several extracolonic malignancies including ovarian, gastric, small bowel, biliary tract, pancreatic, and urinary tract cancers (2, 4). Cancer risk in LS carriers is influenced by a range of factors including age, sex, and the specific MMR gene mutated, with *MLH1* and *MSH2* variant carriers considered at higher risk (5, 6).

Guidelines recommend intensive surveillance, particularly with regular colonoscopy starting in adults as soon as LS is diagnosed and continued typically at 1- to 2-year intervals (7, 8). These surveillance strategies aim to detect colorectal cancer at early stages and remove premalignant lesions—namely adenomas and advanced adenomas (AA), the latter considered at higher risk for progression to cancer. Emerging evidence has raised concerns about the effectiveness of current surveillance programs in reducing colorectal cancer incidence and suggests that shorter colonoscopy intervals may improve early-stage detection (9, 10). Nevertheless, an intensive surveillance program is not easily accepted by patients and is also associated with high healthcare costs. There is therefore high medical need for interventions that could reduce the cancer incidence in these patients.

In LS, the precancer-to-cancer sequence is accelerated because of the loss of MMR function, typically via a somatic “second hit” inactivating the wild-type allele (11). The deficiency in the MMR system is characterized by mutations in microsatellite regions that when occurring in coding regions can result in the formation of recurrent frameshift peptides (FSP) expected to be safe and potent neoantigens (12). Nous-209 immunotherapy is a cancer vaccine comprising 209 shared FSPs selectively enriched in MSI tumors (13). Nous-209 is currently under clinical investigation as preventive measure in LS carriers as stand-alone treatment (14, 15).

Here, we characterize from the molecular point of view precancer lesions in LS carriers to determine the potential of Nous-209 immunotherapy to prevent colorectal cancer by targeting benign lesions during their growth.

## Materials and Methods

### Study design and patient population

This is a noninterventional, nonrandomized observational retrospective study conducted in LS carriers under active surveillance at Istituto di Ricovero e Cura a Carattere Scientifico (IRCCS) Humanitas Research Hospital, Rozzano (Milan, Italy). Ethical approval was obtained from the Institutional Review Board of IRCCS Humanitas Research Hospital prior to study initiation. All procedures involving human participants were conducted in accordance with the ethical standards of the institutional and national research committees and with the principles of the Declaration of Helsinki. Eligible patients were informed about the study and enrolled following the provision of written informed consent. This study did not involve the use of animals in any experimental procedures.

Inclusion criteria were (i) adults (≥18 years) with confirmed germline pathogenic variants in *MLH1* or *MSH2*; (ii) undergoing scheduled colonoscopy during the surveillance program; and (iii) with at least 1 adenomatous or serrated precancerous lesion identified during the colonoscopy. Both LS previvors (patients without the previous history of cancer) and survivors (patients with the previous history of cancer) were included. In this study, survivors were defined as LS carriers with a documented history of a previous Lynch-associated malignancy (e.g., colorectal cancer: *n* = 13; endometrial cancer: *n* = 2). In contrast, non–Lynch-associated tumors such as nonmelanoma skin cancers or microinvasive cervical lesions were not considered. Exclusion criteria included inability to provide consent or inadequate tissue for the molecular analyses.

AAs were defined by specific features, including size (≥10 mm) and/or the presence of a villous component (more than 25%), and/or high-grade dysplasia. Adenomas were grouped according to their size ≤5 or 5 to 10 mm (16).

The final study cohort consisted of 26 carriers, for a total of 62 lesions, all successfully tested by immunohistochemistry (IHC) to determine their MMR status. Residual material after IHC was available for all MMR-deficient (dMMR) samples (39) and for 21 of 23 MMR-proficient (pMMR) specimens. No enrolled participants withdrew consent.

### Sample collection and MMR status assessment

Formalin-fixed, paraffin-embedded (FFPE) specimens, collected from 2020 to 2025, were retrieved from the pathology archives. The MMR status of each lesion was determined by IHC for the 4 MMR proteins (MLH1, RRID: AB_3669002; PMS2, RRID: AB_3669003; MSH2, RRID: AB_2936886; MSH6, RRID: AB_2936885) using VENTANA MMR RxDx Panel Kit, according to the provider’s instructions.

All IHC slides were reviewed by an experienced gastrointestinal pathologist as part of routine diagnostic evaluation, and loss of nuclear staining in tumor cells, with retained staining in adjacent normal epithelium, was interpreted as dMMR.

### MSI testing and Nous-209 mutation identification

All lesions with residual material after MMR testing were shipped to CeGaT GmbH for whole-exome sequencing (WES). A subset of samples was excluded from sequencing analysis because of low tumor cell content as determined by pathology review or insufficient DNA yield after extraction or library preparation failure. Genomic DNA was isolated from FFPE blocks using the MagMax FFPE DNA/RNA Ultra Kit (Thermo Fisher Scientific) according to the manufacturer's instructions. Library preparation was performed using the Twist Human Core Exome + RefSeq + Mitochondrial Panel (Twist Bioscience). Sequencing was carried out on the Illumina NovaSeq 6000 platform (RRID: SCR_016387) using a 2 × 101 bp paired-end read configuration. Demultiplexing of the raw sequencing reads was performed using Illumina bcl2fastq software (version 2.20). Sequencing data analysis was conducted using the Illumina DRAGEN Bio-IT platform (version 4.2.4). Reads were aligned to the human reference genome GRCh38, and duplicate reads were marked during processing.

MSI status was determined using MSIsensor2 (https://github.com/niu-lab/msisensor2), and samples were classified as MSI-high (MSI-H) or MSI-low (MSI-L) lesions as previously described (12).

The analysis pipeline included indexing the GRCh38/hg38 reference genome and scanning 2,793 microsatellite loci. These loci were selected based on their ability to effectively discriminate between microsatellite-stable (MSS) and MSI samples, as determined by a model trained on The Cancer Genome Atlas data. MSI scores were calculated as the proportion of unstable loci among the total analyzed. All analyses were conducted using the default parameters of MSIsensor2. Nonsynonymous tumor mutational burden (TMB) was computed using the Illumina DRAGEN bioinformatics pipeline. The Nous-209 mutations were identified from aligned sequencing data (BAM files) using a lookup-based approach consistent with previously established criteria (13).

### Statistical analysis

The aim of the retrospective study was to establish an association between the presence of Nous-209 mutations and the MMR status of the precancer lesion. Based on previous data, the expected prevalence of dMMR precancer lesions was approximately 30% (12). Therefore, a sample size of 62 precancerous lesions was determined to be adequate to obtain approximately 19 dMMR specimens for the analyses, providing an acceptable level of precision for the descriptive objectives of the study. Notably, the final analysis included 39 dMMR lesions, exceeding the initial expectations and further improving the precision of the analyses.

All statistical analyses and data visualizations were performed using R software (version 4.4.2; RRID: SCR_001905). Comparisons between groups for continuous variables were conducted using the Wilcoxon rank-sum test, whereas the Fisher exact test was used for categorical variables. Correlations between continuous variables were assessed using the Spearman rank correlation coefficient. All statistical tests were two-tailed, and *P* values <0.05 were considered statistically significant.

## Results

### Study cohort and precancer lesion characteristics

**Figure 1.**
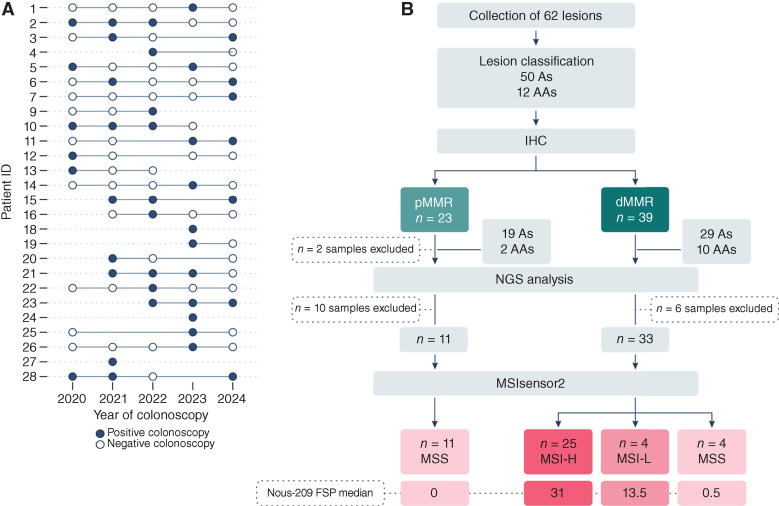
Cohort overview and sample flow chart. **A,** Longitudinal timeline of colonoscopy surveillance across the study cohort. Each row represents an individual patient, and each dot corresponds to a colonoscopy performed over time. White circles indicate colonoscopies negative for lesions, whereas colored circles indicate the presence of at least 1 precancerous lesion. **B,** Study flow chart summarizing the study procedures. A total of 62 lesions were collected, including 50 adenomas (A) and 12 AAs. All lesions were assessed by IHC for MMR protein expression. Thirty-nine dMMR and 21 of 23 pMMR lesions had residual material for NGS. Additional 6 dMMR lesions were excluded: 3 for tumor content lower than 30% and 3 for low DNA concentration after extraction. Additional 10 pMMR lesions were excluded: 8 for low DNA concentration after extraction and 2 for library preparation failure. Overall 33 dMMR and 11 pMMR precancer lesions were sequenced, and the MSI status was assessed using MSIsensor2.

Over a 5-year surveillance period at IRCCS Humanitas Research Hospital, 26 (14 female and 12 male) LS carriers were identified with at least 1 precancerous colorectal lesion during routine colonoscopy (Fig. 1A). Of the total 26 carriers, 16 contributed lesions from a single colonoscopy, 4 provided samples from 2 colonoscopies, and additional 6 carriers contributed with lesions from 3 separate procedures, with a median number of 1.4 lesion/colonoscopy (range, 1–4). The study cohort was composed of participants carrying either *MLH1* (*n* = 10; 38%) or *MSH2* (*n* = 16; 62%) germline mutations. Overall, we observed a similar distribution in terms of age between *MLH1* and *MSH2* carriers and in terms of survivors (*MLH1*: *n* = 6; 60% vs. *MSH2*: *n* = 9; 56%) and previvors (*MLH1*: *n* = 4; 40% vs. *MSH2*: *n* = 7; 44%). Detailed information on the patient characteristics is provided in Table 1. As the study was focused on lesion-level molecular characterization, detailed clinical histories of previous cancers were not extracted from medical records. The study flowchart (Fig. 1B) shows the study procedures and summarizes the results.

**Table 1.** Clinical and pathologic characteristics of the study cohort and corresponding precancerous lesions.

Characteristic	Total lesions *n* = 62 (26 patients)
Age at first colonoscopy, years (mean ± SD)	52 ± 14
Sex, *n* (%)	​
Female	14 (53.8%)
Male	12 (46.2%)
Size of lesion (mm; median ± SD)	5 ± 6
Number of lesion per colonoscopy (mean; min; max)	1.4; 1; 4
MMR germline variant, *n* (%)	​
*MLH1*	10 (38%)
*MSH2*	16 (62%)
MMR status (IHC), *n* (%)	​
pMMR	23 (37%)
dMMR	39 (63%)
IHC MMR gene loss (dMMR; *n* = 39), *n* (%)	​
*MLH1*–*PMS2*	19 (49%)
*MSH2*–*MSH6*	16 (41%)
*MSH6*	2 (5%)
*PMS2*	2 (5%)
Sample type	​
AA (*n* = 12)	​
pMMR	2
dMMR	10
Adenoma (*n* = 50)	​
pMMR	21
dMMR	29
Location, *n* (%)	​
Cecum	5 (8%)
Ascending	14 (23%)
Transverse	18 (29%)
Descending	7 (11%)
Sigma	7 (11%)
Rectum	11 (18%)
Dysplasia grade, *n* (%)	​
Low	59 (95%)
High	3 (5%)
Villous component, *n* (%)	​
Yes	3 (5%)
No	59 (95%)
History of cancer (*n* = 26), *n* (%)	​
Survivor	15 (57.7%)
Previvor	11 (42.3%)

### MMR status and MSI phenotype in precancer lesions

**Figure 2.**
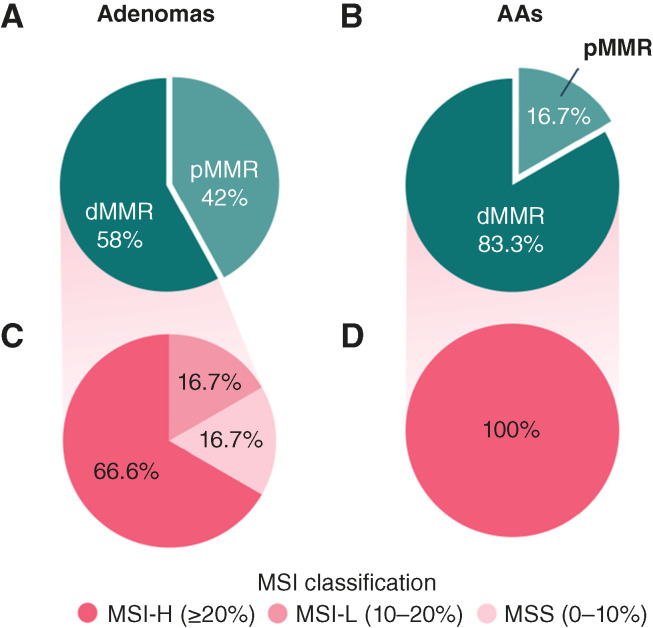
Distribution of MMR status and MSI subtypes in adenomas and AAs. **A,** Pie chart showing the distribution of pMMR and dMMR cases among adenomas. **B,** Pie chart illustrating the proportion of pMMR and dMMR cases in AAs. **C,** Pie chart represents the proportion of dMMR adenomas classified as MSI-H, MSI-L, and MSS according to MSIsensor2. **D,** Pie chart represents the proportion of dMMR AAs classified as MSI-H, MSI-L, and MSS according to MSIsensor2.

Of the 50 non-AAs, IHC revealed 21 of 50 (58%) pMMR and 29 of 50 (42%) dMMR. Of the 12 AAs, 2 of 12 (16.7%) lesions were pMMR and 10 of 12 (83.3%) lesions were dMMR (Fig. 2A and B).

MSI status could be analyzed in 33 of 39 (85%) dMMR specimens, including 24 non-AAs and 9 AAs, and in 11 of 23 pMMR lesions (Fig. 1B). All dMMR AAs were confirmed to be MSI-H; in contrast, among dMMR non-AAs, 66.6% were classified as MSI-H, 16.7% were MSI-L, and the remaining 16.7% were found to be MSS (Fig. 2C and D). All pMMR specimens sequenced were, as expected, classified as MSS.

## Presence of Nous-209 immunotherapy–targeted mutations in precancerous lesions

**Figure 3.**
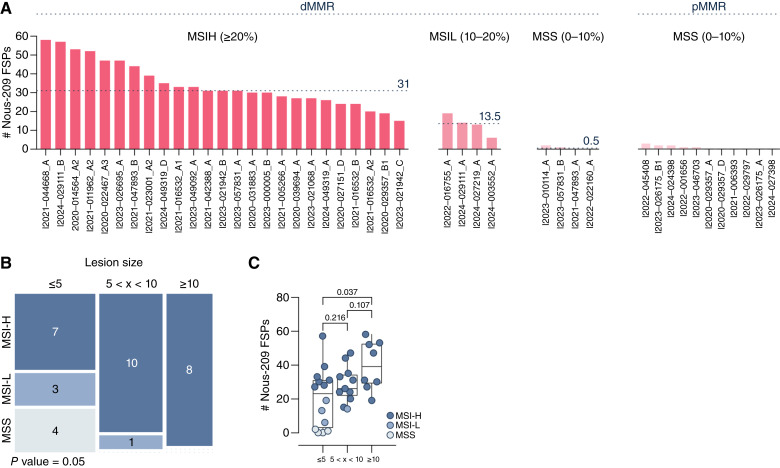
Number of mutations encoded by Nous-209 present in MSI-H precancer lesions and association with lesion size*.***A,** Number of Nous-209 vaccine mutations found in precancer lesions. Bar plot shows the number of Nous-209 mutations detected in each dMMR and pMMR lesion. Lesions are stratified according to their MSI status. Dashed horizontal lines indicate the median number of mutations within each group (MSI-H, MSI-L, MSS/dMMR, and MSS/pMMR). **B,** Mosaic plot showing the distribution of dMMR samples stratified by lesion size (≤5 mm, 5 < x < 10 mm, and ≥10 mm) and classified according to MSIsensor2. **C,** Boxplot displaying the number of Nous-209 mutations across the three lesion size categories. Individual data points represent single samples and are color-coded according to their MSI classification as determined by MSIsensor2.

To characterize the mutational landscape targeted by Nous-209 immunotherapy, we investigated the presence of the Nous-209 mutations in precancerous lesions. We grouped samples according to the MSI status as determined by MSIsensor2. We found a median of 31 mutations in precancer lesions classified as MSI-H (min 15–max 57; Fig. 3A) Interestingly, the 4 cases classified as MSI-L had a median of 13.5 frameshift mutation (min 6–max 19; Fig. 3A), whereas those classified as MSS had a median of 0.5 mutations (min 0–max 2; Fig. 3A), similar to the median found in pMMR lesions (min 0–max 3; Fig. 3A). In line with these results, we found an increased TMB in MSI-H lesions compared with MSI-L and with MSS lesions (Supplementary Fig. S1). The dMMR status was associated with the lesion size, being more frequent in precancer lesions ≥10 mm and in those measuring 5 to 10 mm than in adenoma ≤5 mm in size (Supplementary Fig. S2). Moreover, among dMMR lesions, the MSI status was also associated with the size of the lesions. MSI-H status was more frequent in precancer lesions ≥10 mm and in those measuring 5 to 10 mm in size (Fig. 3B) than in dMMR adenoma ≤5 mm, in which only 50% were MSI-H.

Interestingly, the number of Nous-209 mutations was not significantly different across the three categories of size (Fig. 3C).

Additionally, no statistically significant difference in Nous-209 mutation burden was observed between lesions from LS survivors and previvors (Supplementary Fig. S3).

## Discussion

This work represents the first attempt to provide integrated MMR, MSI, and vaccine-targeted neoantigen characterization of precancerous lesions in LS carriers. We investigated the MMR status by using the standard IHC approach in 62 precancer lesions, 50 adenomas, and 12 AAs collected during routine surveillance in *MLH1* or *MSH2* carriers. dMMR status was found in 58% of adenomas and 83.3% of AAs in this LS cohort. This frequency is slightly higher than the one previously described, suggesting that the prevalence of the dMMR events in precancer lesions may be underestimated in the current literature (17).

MSI status was determined by next-generation sequencing (NGS) analysis according to MSIsensor2. Concordance between MMR and MSI results was then assessed. The two tests are known to provide highly concordant results in metastatic colorectal cancer, in which either of the two can be used to guide immune checkpoint inhibitor treatment decision (18). Moreover, our previous data found 98% concordance between dMMR determined by IHC and MSI-H evaluated with MSIsensor2 in a cohort of incident colorectal cancer in LS carriers (19).

Here, we observed that the concordance between dMMR and MSI-H is very high for AA (100%) but substantially lower in adenoma (66%).

Indeed, 4 dMMR adenomas had an intermediate MSI phenotype corresponding to MSI-L, with a limited number of loci affected by mutations, and additional 4 dMMR adenomas were classified as MSS. Our results indicate that dMMR/MSI-H is more frequent in AAs and in adenomas >5 mm. These findings likely reflect the molecular heterogeneity of the tumorigenic process in LS-associated lesions. Two distinct pathways leading to the formation of MSI colorectal cancer have been described. The first involves the early emergence of MMR deficiency within colonic crypts, whereas the second follows a stepwise progression in which a lesion initially develops as MSS adenoma and subsequently acquire the defect in the MMR system. Data presented in this article are supportive for the presence of both pathways with dMMR/MSI-H adenoma <5 mm potentially emerging from deficient crypts.

**Figure 4.**
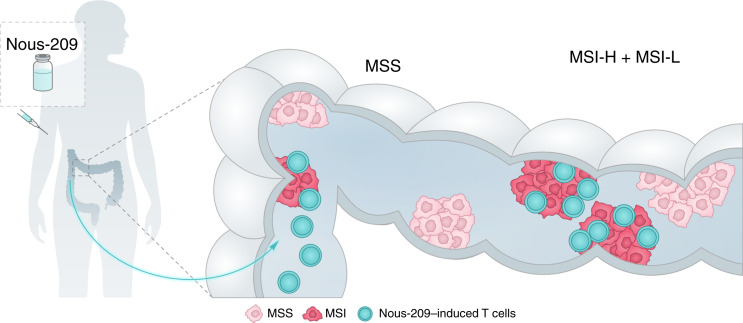
Nous-209 immunotherapy mechanism of action. Nous-209–induced T cells have the potential to target the adenoma–carcinoma sequence in LS carriers by targeting MSI precancer lesions.

We previously established the presence of the Nous-209 mutations in MSI-H metastatic colorectal and endometrial cancers (15, 20). More recently, we published data on their presence in colorectal cancer incident and in urothelial MSI cancers of patients with LS (19). In this study, we established that the mutations selected for Nous-209 immunotherapy based on their expression in late-stage colorectal cancer actually arise early in carcinogenesis, as soon as MSI is acquired. MSI-H precancer lesions harbor a median of 31 FSPs producing neoantigens potentially targeted by Nous-209 immunotherapy. Interestingly, the few small adenomas (≤5 mm) classified as MSI-H show a number of Nous-209 mutations comparable with those observed in larger adenomas and in AAs. Although this should be considered a preliminary observation due to the limited number of dMMR/MSI-H samples ≤5 mm in size analyzed (*n* = 7), it suggests that Nous-209 mutations may arise very early in the development of MSI-H lesions. A schematic representation summarizing the study findings is shown in Fig. 4.

Our findings are consistent with a stepwise acquisition model, although causality cannot be inferred from this retrospective dataset. Moreover, our results strengthen the biological rationale for immunoprevention but also support prevention trial design in which reduction in MSI-H adenomas could serve as a clinically meaningful surrogate endpoint for Nous-209 efficacy. Although we acknowledge that this is a preliminary study relying on a limited set of samples collected retrospectively in a single clinical center, it represents an important advancement in our understanding of precancer lesion evolution and the potential role of immunoprevention in LS.

## Supplementary Material

Supplementary Figure S1 Supplementary Figure S1. Boxplot showing tumor mutational burden (TMB) stratified by molecular subtype: dMMR/MSI-H, dMMR/MSI-L. dMMR/MSS. and oMMR/MSS.

Supplementary Figure S2 Supplementary Figure S2. Mosaic plot illustrating the distribution of samples according to IHC MMR status (dMMR vs. pMMR) across lesion size categories (≤5 mm. 5<x<10mm. and ≥10mm).

Supplementary Figure S3 Supplementary Figure S3. No statistically significant difference in Nous-209 mutation burden was observed between lesions from Lynch syndrome survivors and previvors.

## Data Availability

The WES data generated in this study are available through the European Genome-Phenome Archive under accession number EGAS50000001546. All other data are available from the corresponding author upon reasonable request.
