# Supplementary figures and images for: Mutations Targeted by Nous-209 Immunotherapy Occur Early in Lynch Syndrome Carriers’ Precancer Lesions with Microsatellite Instability
Source: Cancer Prev Res (Phila). 2026 May 8;19(6):361–7. doi: 10.1158/1940-6207.CAPR-25-0388 (PMC13223544; doi:10.1158/1940-6207.CAPR-25-0388)

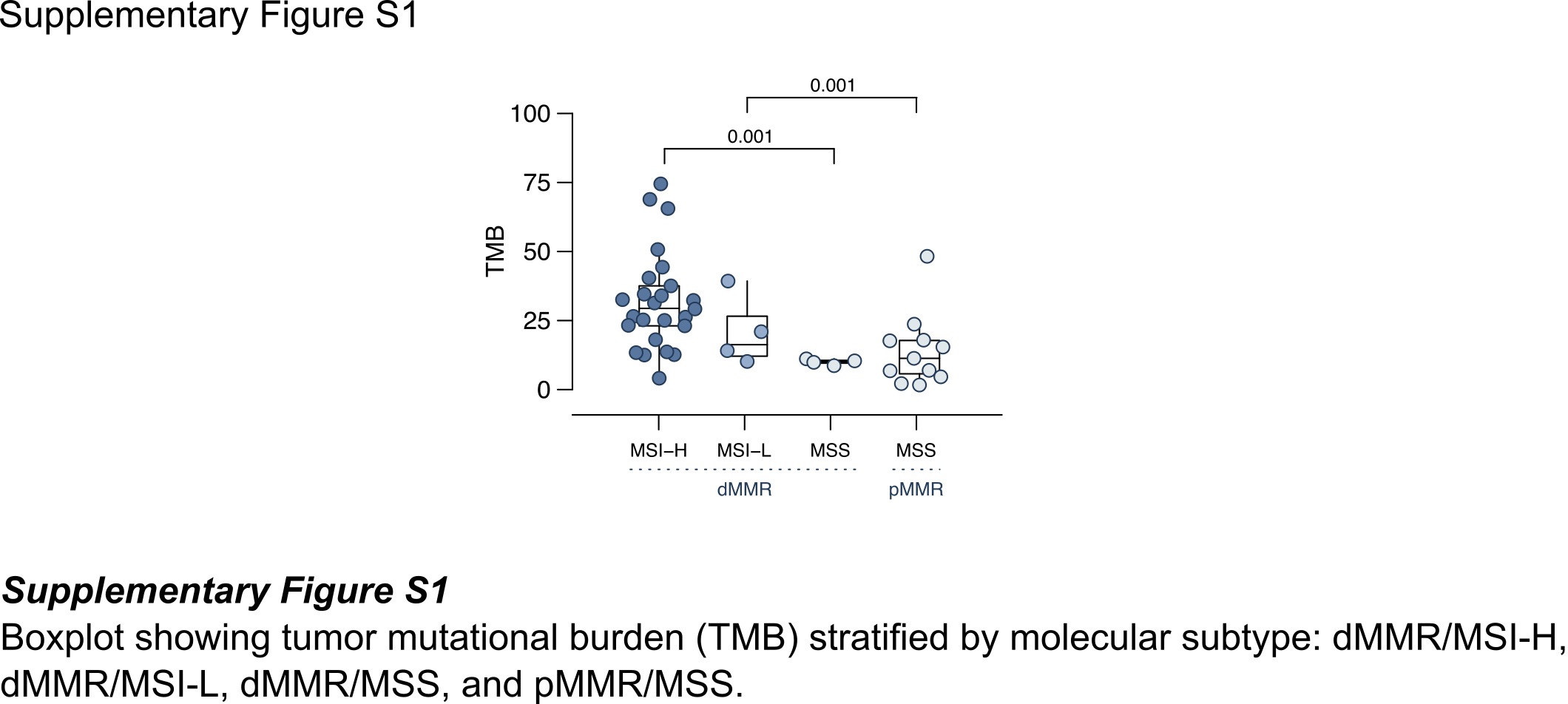

Supplement: Supplementary Figure S1 — Boxplot showing tumor mutational burden (TMB) stratified by molecular subtype: dMMR/MSI-H, dMMR/MSI-L. dMMR/MSS. and oMMR/MSS. [file capr-25-0388_supplementary_figure_s1_suppsf1.png]

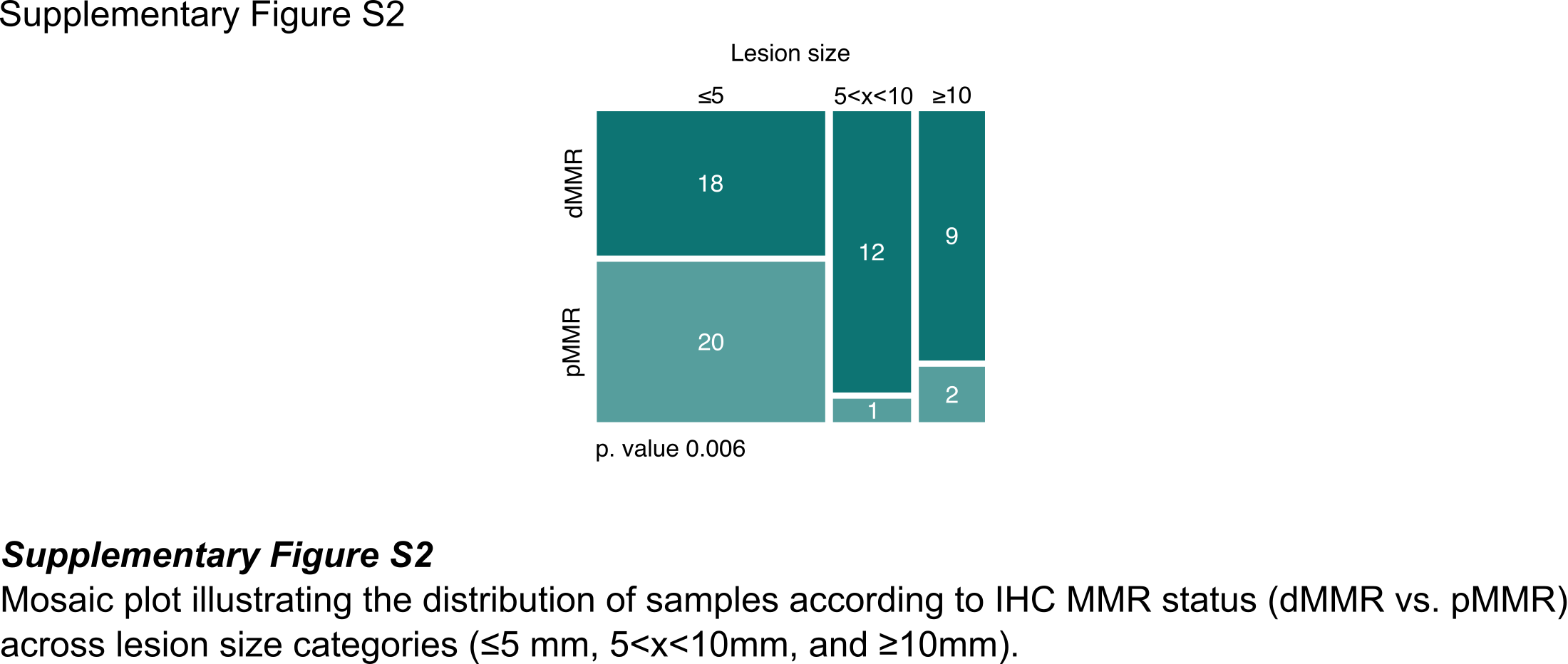

Supplement: Supplementary Figure S2 — Mosaic plot illustrating the distribution of samples according to IHC MMR status (dMMR vs. pMMR) across lesion size categories (≤5 mm. 5<x<10mm. and ≥10mm). [file capr-25-0388_supplementary_figure_s2_suppsf2.png]

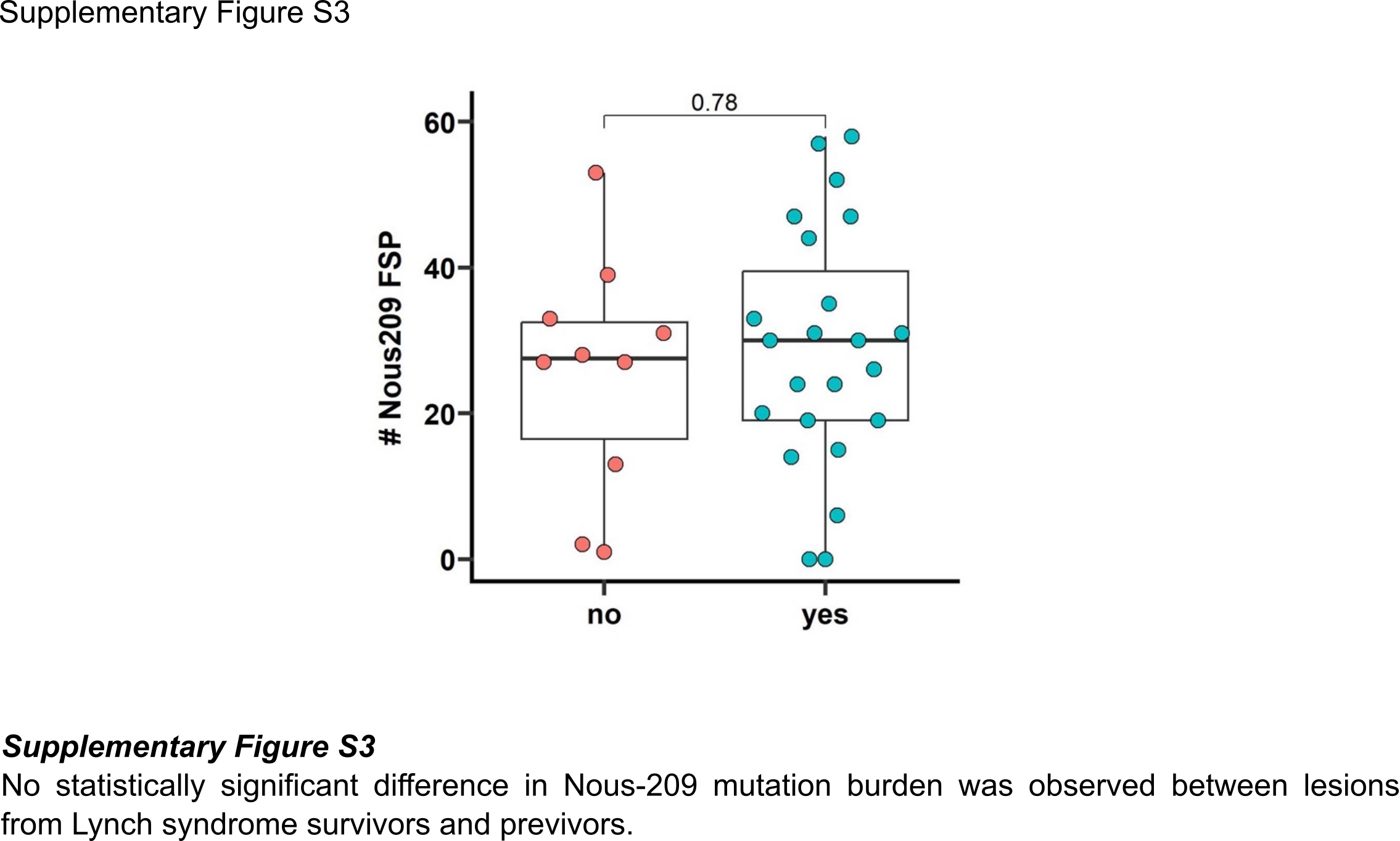

Supplement: Supplementary Figure S3 — No statistically significant difference in Nous-209 mutation burden was observed between lesions from Lynch syndrome survivors and previvors. [file capr-25-0388_supplementary_figure_s3_suppsf3.png]
